# Multi-Spectral Radiation Temperature Measurement: A High-Precision Method Based on Inversion Using an Enhanced Particle Swarm Optimization Algorithm with Multiple Strategies

**DOI:** 10.3390/s24186003

**Published:** 2024-09-17

**Authors:** Xiaodong Wang, Shuaifeng Han

**Affiliations:** College of Computer and Information Engineering, Henan Normal University, Xinxiang 453007, China; mhanshuaifeng@163.com

**Keywords:** multi-spectral thermometry, temperature inversion, spectral emissivity model, optimization algorithm, data processing

## Abstract

Multi-spectral temperature measurement technology has been found to have extensive applications in engineering practice. Addressing the challenges posed by unknown emissivity in multi-spectral temperature measurement data processing, this paper adds emissivity constraints to the objective function. It proposes a multi-spectral radiation temperature measurement data processing model realized through a particle swarm optimization algorithm improved based on multiple strategies. This paper simulates six material models with distinct emissivity trends. The simulation results indicate that the algorithm calculates an average relative temperature error of less than 0.3%, with an average computation time of merely 0.24 s. When applied to the temperature testing of silicon carbide and tungsten, experimental data further confirmed its accuracy: the absolute temperature error for silicon carbide (tungsten) is less than 4 K (7 K), and the average relative error is below 0.4% (0.3%), while two materials maintain an average computation time of 0.33 s. In summary, the improved particle swarm optimization algorithm demonstrates strong performance and high accuracy in multi-spectral radiation thermometry, making it a feasible solution for addressing multi-spectral temperature measurement challenges in practical engineering applications. Additionally, it can be extended to other multi-spectral systems.

## 1. Introduction

Multi-spectral radiation temperature measurement methods are widely used in various fields, such as rocket engine performance evaluation, gas turbine material testing, and metal smelting temperature monitoring [1,2,3,4], due to their rapid response, noncontact nature, and unlimited temperature measurement range [5,6,7,8]. The technology possesses the capability to simultaneously determine the true temperature and spectral emissivity of materials. Leveraging the multi-spectral channel data provided by this technology, this paper establishes *N* radiation equations that encompass N+1 unknown parameters. However, these equations are underdetermined due to the uncertainty of emissivity, making them difficult to solve [9,10].

Traditional methods for temperature measurement often rely on assumptive models based on fixed relationships between emissivity and wavelength or temperature. These assumptive models can be linear or nonlinear, with the aim of transforming complex radiation equations into solvable forms. Subsequently, mathematical techniques, including the least squares method, are employed to solve the transformed equations. The objective is to concurrently determine the temperature and emissivity at two successive temperature measurement points [11,12,13]. However, in practical applications, the spectral emissivity of the target object often varies inconsistently at different measurement locations, making it difficult for methods based on fixed assumptive models to obtain accurate results. To improve the accuracy and reliability of temperature measurements, the field of multi-spectral thermometry needs to explore more flexible and adaptive methods to handle these variations.

Traditional methods for temperature measurement often rely on assumptive models. As artificial intelligence technology continues to advance, neural networks have emerged as a potent tool for tackling nonlinear mapping problems in radiation temperature measurement. Sun et al. successfully processed multi-wavelength radiation temperature data by utilizing the traditional backpropagation (the following will be referred to as BP) neural network [14]. Xi et al. constructed an infrared radiation model based on the Radial Basis Function (the following will be referred to as RBF) neural network, achieving an accurate estimation of target spectral emissivity [15]. Chen et al. further delved into a high-temperature multi-wavelength temperature measurement method that is based on an adaptive emissivity model. This approach is widely applicable to most engineering materials, as it does not rely on models where emissivity is a function of the wavelength [16]. However, the BP neural network exhibits certain limitations, including susceptibility to local minima, sluggish convergence, and inadequate generalization ability, which render it challenging to satisfy practical application requirements. In recent years, transformer neural networks and long sequence time series prediction (the following will be referred to as LSTM) have demonstrated excellent prediction performance [17,18]. Xing et al. proposed the generalized inverse matrix long short-term memory (the following will be referred to as GIM-LSTM) data processing algorithm, which combines the advantages of the generalized inverse matrix and LSTM neural network, achieving the efficient and accurate processing of multi-wavelength pyrometry (the following will be referred to as MWP) data [19]. However, the training of neural network models necessitates a substantial amount of valid data samples and considerable time for preprocessing these data samples prior to training. Therefore, in industrial applications where real-time performance and accuracy are required, machine learning methods currently need to be improved for implementation.

In recent years, some new methods have emerged in the field of multi-spectral radiation temperature measurement that do not rely on emissivity models or data samples. These innovative approaches convert data processing into optimization problems, aiming to minimize the deviation between the calculated temperature derived from various spectral channels and the actual temperature. Xing et al. experimented with different optimization algorithms, specifically, gradient projection and internal penalty function algorithms. They found that the internal penalty function algorithm performed better, achieving a maximum error of 25K but with a time consumption of 210 s [20]. Liang et al. employed the Generalized Inverse Matrix and External Penalty Function (the following will be referred to as GIM-EPF) method to estimate the temperature of a rocket nozzle, achieving a maximum error of 0.65% and a calculation time of merely 3.4 s. [21]. K. Yu et al. employed an iterative algorithm for optimization, achieving an error of less than 0.5% and a calculation time of 0.2 s [22]. Tian et al. innovatively proposed two optimization algorithms. Two methods, Sequential Random Coordinate Shrinkage (the following will be referred to as SRCS) and Multi-Population Genetic (the following will be referred to as MPG), were considered. Notably, the MPG algorithm requires only 0.68 s for inversion and achieves an error rate of 0.4% [23]. However, it should be noted that even if a near-optimal solution is found, the temperature calculation error may still be significant. Nevertheless, previous studies have shown that the objective function in multi-spectral temperature measurement has multiple local minima. Even if the function optimization result is close to the optimal value of 0, the calculation error of the temperature may still be large, meaning that the optimal function result does not guarantee the effectiveness of the temperature calculation. Therefore, although the algorithm may find an effective optimization result, the actual temperature calculation may need to be revised.

To address the issue of the objective function easily falling into local extrema and to achieve the simultaneous and accurate inversion of true temperature and spectral emissivity, we introduced relevant constraints on emissivity based on the deviation between the calculated temperature and actual temperature, under three different relationships between detection signals and reference signals. This led to the development of a novel multi-spectral radiation temperature measurement data processing model. To optimize this model, we chose the particle swarm optimization (the following will be referred to as the PSO) algorithm, which is particularly suited for functions with multiple local extrema. Furthermore, we enhanced the PSO algorithm by adopting the multi-strategy particle swarm optimization (the following will be referred to as MSPSO) approach. This enhancement aims to improve its global search capabilities and ability to escape local extrema. Subsequently, we validated the effectiveness of this data processing method by simulating six hypothetical materials with different emissivity distributions and using actual measurement data from silicon carbide and tungsten. This achievement not only improves the accuracy of the temperature measurement but also demonstrates the potential value of the multi-spectral temperature measurement technology.

## 2. Multi-Spectral Temperature Measurement Principle

### 2.1. Reference Temperature Mode

In accordance with Planck’s radiation law, the output signal $V_{i}$ of each channel, denoted as the *i*th channel, can be derived when using a multi-spectral radiation thermometer with *n* wavelength channels:

(1) $$V_{i}=A_{\lambda_{i}}\varepsilon \left(\lambda_{i},T\right)\frac{1}{\lambda_{i}^{5}\left(e^{C_{2}/\lambda_{i}T}-1\right)}\left(i=1,2,3,\ldots ,n\right),$$

In the given formula, $A_{\lambda_{i}}$ is a constant that is related only to the wavelength and independent of temperature. This constant is influenced by factors such as the detector’s spectral responsivity, the transmittance of optical elements, the instrument’s geometry, and the second radiation constant $C_{2}$. The spectral emissivity of the material at its true temperature *T* is represented by the variable $\varepsilon \left(\lambda_{i},T\right)$, where $\lambda_{i}$ denotes the effective wavelength of the *i*th spectral channel. Together, these parameters govern the output signal of the multi-spectral radiation thermometer.

Under specific conditions, when $C_{2}/\left(\lambda_{i}T\right)<<1$, the original complex Equation (1) can be simplified using the Wien approximation as follows:

(2) $$V_{i}=A_{\lambda_{i}}\varepsilon \left(\lambda_{i},T\right)\lambda_{i}^{-5}e^{-\frac{c_{2}}{\lambda_{i}T}}\left(i=1,2,3,\ldots ,n\right),$$

At the blackbody reference temperature $T^{\prime }$, the output signal of the *i*th spectral channel can be represented as $V_{i}^{\prime }$ as shown below:

(3) $$V_{i}^{\prime }=A_{\lambda_{i}}\varepsilon \left(\lambda_{i},T^{\prime }\right)\lambda_{i}^{-5}e^{-\frac{c_{2}}{\lambda_{i}T}}\left(i=1,2,3,\ldots ,n\right),$$

In the formula, $\varepsilon \left(\lambda_{i},T^{\prime }\right)$ represents the emissivity of the blackbody at a specific wavelength $\lambda_{i}$ and temperature $T^{\prime }$, which is generally set to 1.

By taking the ratio of Equations (2) and (3) mentioned above, we can construct a reference temperature model:

(4) $$\frac{V_{i}}{V_{i}^{\prime }}=\varepsilon \left(\lambda_{i},T\right)e^{\frac{c_{2}}{\lambda_{i}}\left(\frac{1}{T^{\prime }}-\frac{1}{T}\right)},$$

This model is uniquely designed, utilizing a blackbody as a reference, which eliminates the need for constants and simplifies the operational process. Moreover, it necessitates only the measurement of the voltage output from each channel at any chosen reference temperature, thereby facilitating a relatively straightforward calibration process. It is important to note that the selection of the reference temperature does not impact the final calculation results. However, in the process of the multi-spectral radiation temperature measurement, we always face one more unknown parameter than the number of equations. Therefore, this paper adopts a new strategy to transform the problem of solving this underdetermined system of equations into a constrained optimization problem. Utilizing the reference temperature model as a foundation, we further develop a constrained optimization model tailored for multi-spectral data processing.

### 2.2. Constrained Optimization Model for Multi-Spectral Temperature Measurement Data Processing

Constrained optimization problems not only require optimizing the objective function to find the optimal solution but also need to satisfy specific constraint conditions. These constraints can be equations or inequalities, aiming to restrict the value range of variables or ensure that the optimization results meet certain requirements. In this paper, our goal is to find the minimum value that satisfies all constraint conditions, which can be formulated as:

(5) $$\left\{\begin{array}{c} \min f(x)\\ Ax\geq b \end{array}\right.,$$

In this formula, *x* represents the variable we want to optimize, also known as the decision variable. *A* is the coefficient matrix of the constraints, and *b* is the vector associated with these constraints.

Equation (4) shows that, given the emissivity $\varepsilon \left(\lambda_{i},T\right)$, the temperatures in each spectral channel are consistent, and this temperature is the actual temperature. Therefore, theoretically, there should be no deviation in the temperatures calculated from each channel, leading to the following formula:

(6) $$\frac{1}{n}\sum_{i=1}^{n}\frac{T_{i}^{2}}{E^{2}\left(T_{i}\right)}=1,$$

In the above formula, $T_{i}$ represents the temperature calculated from each spectral channel, and its expression can be described as:

(7) $$T_{i}=\frac{1}{\frac{1}{T^{\prime }}+\frac{\lambda_{i}}{C_{2}}\left[\ln\varepsilon \left(\lambda_{i},T\right)-\ln\left(\frac{V_{i}}{V_{i}^{\prime }}\right)\right]},$$

$E\left(T_{i}\right)$ represents the average value of the temperature calculations from all spectral channels, which can be expressed as:

(8) $$E\left(T_{i}\right)=\frac{1}{n}\sum_{i=1}^{n}T_{i},$$

Due to the unknown emissivity, we can transform Equation (6) into an optimization problem based on the principle of minimizing deviation, striving to make the temperature deviation as close to zero as possible:

(9) $$\min F=\left|\left(\frac{1}{n}\sum_{i=1}^{n}\frac{T_{i}^{2}}{E^{2}\left(T_{i}\right)}\right)-1\right|\to 0,$$

The emissivity of all materials is between 0 and 1. Therefore, the range of emissivity values can be expressed as follows:

(10) $$\left\{\begin{array}{cc} \varepsilon \left(\lambda_{i},T\right) & >0\\ -\varepsilon \left(\lambda_{i},T\right) & >-1 \end{array}\right.,$$

By imposing constraints on the emissivity value range, we transform the problem of processing multi-spectral radiation temperature measurement data into an optimization problem. The precise formulation of this scenario is as follows:

(11) $$\left\{\begin{array}{c} \min F=\left|\left(\frac{1}{n}\sum_{i=1}^{n}\frac{T_{i}^{2}}{E^{2}\left(T_{i}\right)}\right)-1\right|\\ \varepsilon \left(\lambda_{i},T\right)>0\\ -\varepsilon \left(\lambda_{i},T\right)>-1 \end{array}\right.,$$

We further transform the objective function. Let $T_{i}$ represent the true temperature, and $E(T_{i})$ represent the average temperature across all channels. The true temperature $T_{i}$ and the average temperature $E(T_{i})$ across all channels can be expressed as follows:

(12) $$T_{i}=\frac{c_{2}}{\lambda_{i}}\cdot \frac{1}{x_{i}+D_{i}},$$

(13) $$E\left(T_{i}\right)=\frac{1}{n}\sum_{i=1}^{n}\frac{c_{2}}{\lambda_{i}}\cdot \frac{1}{x_{i}+D_{i}},$$

where $x_{i}=\ln\varepsilon \left(\lambda_{i},T\right)$, $D_{i}=\frac{c_{2}}{\lambda_{i}T^{\prime }}-\ln\left(\frac{V_{i}}{V_{i}^{\prime }}\right)$.

Then, Equation (9) can be transformed into the following form:

(14) $$\min f\left(x\right)=\sum_{i=1}^{n}{\left(\frac{c_{2}}{\lambda_{i}}\cdot \frac{1}{x_{i}+D_{i}}-\frac{1}{n}\sum_{i=1}^{n}\frac{c_{2}}{\lambda_{i}}\cdot \frac{1}{x_{i}+D_{i}}\right)}^{2},$$

In multi-spectral radiometric measurements, the value of the spectral emissivity lies within the [0, 1] range, and the constraint range for $x_{i}$ does not exceed 0. Therefore, Equation (11) can be rewritten as follows:

(15) $$\left\{\begin{array}{c} \min f\left(x\right)=\sum_{i=1}^{n}{\left(\frac{c_{2}}{\lambda_{i}}\cdot \frac{1}{x_{i}+D_{i}}-\frac{1}{n}\sum_{i=1}^{n}\frac{c_{2}}{\lambda_{i}}\cdot \frac{1}{x_{i}+D_{i}}\right)}^{2}\\ x_{i}\leq 0 \end{array}\right.$$

According to the expression of the objective function mentioned above, we are faced with a typical nondifferentiable nonlinear constrained optimization problem. To solve this problem, an effective optimization algorithm is needed, and this algorithm cannot rely on gradients. The multi-strategy improved particle swarm optimization algorithm employed in this paper is highly suitable for addressing this type of optimization problem.

## 3. Principle of MSPSO Algorithm

Formula (11) of the multi-spectral pyrometer data processing model depicts a complex relationship between the object’s radiation signal and its true temperature, rendering it challenging to directly solve for the true temperature. To tackle this issue, we convert the original problem into a constrained optimization problem. However, since both the true temperature and emissivity are unknown, the radiation equations become underdetermined, potentially leading to multiple local optimal solutions, which increases the difficulty and uncertainty of the solution. Furthermore, the nonlinearity and nondifferentiability of the objective function make traditional gradient-based optimization methods inapplicable. Therefore, we need to adopt a gradient-free optimization algorithm with excellent global search capabilities. Despite the particle swarm optimization algorithm demonstrating strong global search capabilities, it is prone to being trapped in local optimal solutions. In this section, we enhance the particle swarm optimization algorithm using multiple strategies, significantly enhancing its ability to escape local optima and accurately solve the data processing model of the multi-spectral pyrometer. The specific methodology is described as follows.

### 3.1. Principle of PSO Algorithm

The particle swarm optimization (PSO) algorithm, which mimics the foraging behavior of bird flocks, is employed to address multi-dimensional parameter global search optimization problems. In this algorithm, each ‘bird’ or particle represents a potential solution, and it seeks the optimal solution by continually adjusting its flight speed and direction [24]. The movement of particles in the solution space is represented by a velocity vector, and the optimal solution is searched by simulating the update of particle positions and the adjustment of velocities. During the optimization process, the movement of particles is influenced by three factors: inheritance of the previous velocity, self-learning, and information exchange within the population, which are specifically described by the following formulas:

(16) $$v_{i}\left(k+1\right)=\omega v_{i}\left(k\right)+c_{1}r_{1}\left(P_{\mathrm{best}.\;i}\left(k\right)-x_{i}\left(k\right)\right)+c_{2}r_{2}\left(G_{\mathrm{best}.\;i}\left(k\right)-x_{i}\left(k\right)\right),$$

(17) $$x_{i}\left(k+1\right)=x_{i}\left(k\right)+v_{i}\left(k+1\right)),$$

In the formula mentioned above, ω represents the inertia weight coefficient, whereas $c_{1}$ and $c_{2}$ stand for the individual cognition and social cognition factors, respectively. The notations $x_{i}\left(k\right)$ and $v_{i}\left(k\right)$ indicate the position and velocity of the *i*th particle at the *k*th iteration. $r_{1}$ and $r_{2}$ are random numbers used in the computation. $P_{\mathrm{best}.i\;}$ signifies the optimal position achieved by the *i*th particle, and $G_{\mathrm{best}.i\;}$ represents the best position attained by the entire population. Their initial velocity and learning factors influence the search performance of the particles. Specifically, $c_{1}$ governs the particle’s movement towards its individual best position, while $c_{2}$ controls its flight towards the globally optimal position of the population. In practical optimization scenarios, the termination conditions are typically based on achieving the global optimal solution and adaptability. Specifically, in addressing the problem of the multi-spectral radiation temperature measurement, this paper details the process of optimizing the objective function using the particle swarm optimization (PSO) algorithm as follows. Initially, the velocity ($v_{i}$) and particle positions ($x_{i}$) are initialized through Equations (16) and (17). The initialized particle positions are then assigned to the emissivity $\ln\epsilon \left(\lambda_{i},T\right)=x_{i}$, in other words, $x_{i}$ in Equation (15). Then, the fitness value is calculated using the objective function in Equation (15). Subsequently, the velocity and particle positions are iteratively optimized to minimize the fitness value, bringing it closer to zero, thereby obtaining the optimal solution, which is the emissivity.

Before the particle flies, it needs to be checked for overspeed. If the $v_{i}\left(k\right)$ velocity exceeds the limit, it is replaced by the boundary value. After flying, we evaluate whether the position $x_{i}\left(k\right)$ exceeds the search range, and if it does, we replace it with the boundary value. We update the $P_{\mathrm{best}.\;i}$ and $G_{\mathrm{best}\;}$ of the particle swarm based on changes in the fitness values. The formula is as follows:

(18) $$\begin{array}{c} P_{\mathrm{best}.\;i}\left(k+1\right)=\left\{\begin{array}{cc} P_{\mathrm{best}.\;i}\left(k\right), & f\left(x_{i}\left(k\right)\right)\leq f\left(P_{\mathrm{best}.\;i}\left(k\right)\right)\\ x_{i}\left(k_{+1}\right) & f\left(x_{i}\left(k\right)\right)>f\left(P_{\mathrm{best}.\;i}\left(k\right)\right) \end{array}\right.\\ G_{\mathrm{best}\;}\left(k+1\right)=\left\{\begin{array}{cc} G_{\mathrm{best}\;}\left(k\right), & f\left(P_{\mathrm{best}.\;i}\left(k\right)\right)\leq f\left(G_{\mathrm{best}\;}\left(k\right)\right)\\ P_{\mathrm{best}.\;i}\left(k\right), & f\left(P_{\mathrm{best}.\;i}\left(k\right)>f\left(G_{\mathrm{best}\;}\left(k\right)\right)\right. \end{array}\right. \end{array},$$

The particle swarm algorithm sometimes converges too early to a local optimal solution instead of the global optimal solution. This may be because the particles rely too heavily on the best positions found during the search process, preventing them from exploring other areas of the solution space. However, since the objective function in this paper contains multiple local extrema, an optimization algorithm with strong global search capabilities is required. Therefore, the following improvement strategies are proposed in this paper.

### 3.2. Chaotic Opposition-Based Learning Initialization

Random initialization of the population may lead to an uneven distribution of particle swarm individuals, reducing population diversity, which could potentially cause the algorithm to converge too early. In order to tackle this challenge, the present paper combines chaotic mapping and opposition-based learning techniques for initializing the population. Specifically, the Pwlcm piecewise chaotic mapping is initially employed to generate the population [25]. Considering that the Pwlcm demonstrates a uniform density function across its designated interval, the distribution at specific values appears nearly flat. The ergodicity and randomness inherent in this chaotic sequence are advantageous for distributing particle swarm individuals across the entire solution space, ultimately enhancing population diversity. The formula for the Pwlcm mapping is given as follows:

(19) $$x\left(k+1\right)=\left\{\begin{array}{c} \frac{x(k)}{p},0\leq x\left(k\right)<p\\ \frac{x(k)-p}{0.5-p},p\leq x\left(k\right)<0.5\\ \frac{1-p-x(k)}{0.5-p},0.5\leq x\left(k\right)<1-p\\ \frac{1-x(k)}{p},1-p\leq x\left(k\right)<1 \end{array}\right.,$$

where the value range of the control parameter *p* is [0, 1], and in this paper, we will set it to 0.35.

Subsequently, we apply a mapping of the chaotic sequence onto the solution space, yielding a chaotically initialized population. We then perform opposition-based learning on this population as outlined in [26]. The formula for opposition-based learning is presented in Equation (10):

(20) x(k+1)=k×(Ub+Lb)−x(k),

where *k* is a random vector of size 1×d that follows a normal distribution. Lb and Ub represent the lower and upper bounds of the solution space, while x(k) represents the chaotically initialized population.

Finally, we merge the initial population with the opposition population, calculate their fitness, and select the top *N* individuals from them as the new initial population.

### 3.3. Adaptive Particle Swarm Parameter Setting

In the past, setting the values of ω, $c_{1}$, and $c_{2}$ in particle swarm optimization (PSO) algorithms was often based on experience or extensive simulation experiments, and these parameters remained constant during the optimization process. However, if these parameters can be dynamically adjusted during the optimization process, the performance of the PSO algorithm will be improved. Among them, the inertia weight coefficient ω is crucial to the algorithm’s search capability, as it controls the inertia of particles to maintain their original direction of motion. A larger ω value is beneficial for global search but may be disadvantageous for local optimization, while a smaller ω value has the opposite effect. To control the variation, we choose a negative hyperbolic tangent curve within the range of [−4, 4]. This nonlinear control strategy exhibits a gradual decrease in the early stage of the search process, which is beneficial for conducting a global search. In the middle stage, it decreases approximately linearly, enhancing the efficiency of the local search. Furthermore, the rate of change decreases again in the later stage, ensuring precise local exploration and ultimately facilitating the discovery of the global optimal solution. The formula for the inertia weight is provided as follows:

(21) $$\omega =\left(\omega_{\max}+\omega_{\min}\right)/2+\tanh\left(-4+8\times \left(k_{\max}-k\right)/k_{\max}\right)\left(\omega_{\max}-\omega_{\min}\right)/2,$$

where $\omega_{\max}$ = 0.9 and $\omega_{\min}$ = 0.5, with the current iteration number denoted as *k* and the maximum iteration number as $k_{\max}$.

When the value of $c_{1}$ is greater than $c_{2}$, particles are more inclined to move along the individual optimal path; conversely, they may choose the group optimal path. In the early stages of evolution, the difference between individuals and the group should be reduced to avoid stagnation in convergence. In the initial stage, emphasis should be placed on global search, especially on the self-awareness and ergodicity of particles, to reduce the risk of falling into local optima. Therefore, in the early stages, ω and $c_{1}$ need to be relatively large to avoid premature convergence. As iterations progress, it is necessary to strengthen the interaction between particles so that the population’s optimal solution has a greater impact on each particle, enabling particles to converge faster towards the global optimal solution. Therefore, ω and $c_{1}$ will gradually decrease, while $c_{2}$ will gradually increase, achieving a dynamic balance. The specific formula is as follows:

(22) $$c_{1}=c_{1\max}-k\left(c_{1\max}-c_{1\min}\right)/k_{\max},$$

(23) $$c_{2}=c_{2\;\min\;}-k\left(c_{2\min}-c_{2\max}\right)/k_{\max},$$

In the particle swarm optimization (PSO) algorithm, we set the variation ranges for the self-learning factor and the social learning factor. Specifically, we define $c_{1\max}$ = 2.50, $c_{2\max}$ = 1.25, $c_{1\min}$ = 0.25, and $c_{2\min}$ = 2.50. The value of $c_{1}$ decreases linearly from its maximum value of 2.50 to its minimum value of 0.25 as the iteration progresses, while the value of $c_{2}$ increases linearly from 1.25 to 2.50. In the early stages of iteration, this setting augments the global search capability by assigning a higher value to $c_{1}$, which motivates particles to explore more extensively within the search space. As the iteration count rises, $c_{1}$ diminishes while $c_{2}$ escalates, guiding the algorithm towards local search. This shift enhances the precision of locating the optimal solution and hastens the convergence of particles toward the global optimum.

### 3.4. Adaptive Cauchy–Gaussian Mutation Strategy

To address the issue of particle swarm optimization being susceptible to converging to locally optimal solutions, we utilize the Cauchy–Gaussian mutation strategy to perturb the optimal positions of individual particles. In the early stages of algorithm iteration, to maintain population diversity and conduct an extensive search, we use the Cauchy distribution operator, which has strong perturbation capabilities and can enhance global search capabilities. In the later stages of iteration, when the searcher discovers a local optimal position, we introduce the Gaussian distribution operator to prevent population stagnation and avoid falling into local optima. This enhances the local exploration capabilities and aids the algorithm in escaping from local optima. Therefore, this paper designs an adaptive Cauchy–Gaussian hybrid mutation strategy [27]. In the early stages of iteration, emphasis is placed on Cauchy perturbation to maintain diversity, while in the later stages, emphasis is on Gaussian perturbation to assist in escaping local optima. In each iteration, this hybrid mutation is applied to the globally optimal individual, and a better position is selected to replace the original position. The formula for the adaptive Cauchy–Gaussian mutation strategy is as follows:

(24) $$U_{\mathrm{best}\;}^{k}=X_{\mathrm{best}\;}^{k}\left[1+\lambda_{1}\;\mathrm{Cauchy}\;\left(0,1\right)+\lambda_{2}\;\mathrm{Gauss}\;\left(0,1\right)\right],$$

In the aforementioned formula, *k* denotes the current iteration number, with hyperparameters set as $\lambda_{1}=1-k^{2}/K_{\max}^{2}$ and $\lambda_{2}=k^{2}/K_{\max}^{2}$. Here, $K_{\max}$ signifies the maximum number of iterations. $X_{\mathrm{best}\;}$ and $U_{\mathrm{best}\;}$ represent the original position and the mutated new position of the globally optimal individual at the *k*th iteration, respectively. Cauchy(0,1) and Gauss(0,1) are random variables adhering to the standard Cauchy and Gaussian distributions, respectively. $\lambda_{1}$ and $\lambda_{2}$ are adaptive parameters that dynamically adjust according to the iteration count.

The optimal solution obtained through the adaptive Cauchy–Gaussian mutation strategy is not necessarily better than the original solution. Therefore, we adopt a greedy selection strategy [28], where the original solution is only replaced if the fitness calculated by the Cauchy–Gaussian mutation strategy is better. The specific formula is as follows:

(25) $$x{\left(k\right)}^{\prime }=\left\{\begin{array}{cc} x(k), & f\left(x\left(k\right)\right)<f\left(x_{\mathrm{new}\;}\right)\\ x_{\mathrm{new}\;}, & f\left(x\left(k\right)\right)>f\left(x_{\mathrm{new}\;}\right) \end{array}\right.,$$

$x{\left(k\right)}^{\prime }$ represents the position of the particle swarm after selection by the greedy algorithm.

### 3.5. Basic Flow of MSPSO Algorithm

This paper first initializes the particle positions using chaotic opposition-based learning. During iteration, the parameters in PSO are improved and adaptively changed. To enable particles to escape from local optimal solutions, an adaptive Cauchy–Gaussian mutation strategy is incorporated, which generates new individuals and selects high-quality individuals through a greedy strategy to enhance algorithm efficiency. At the same time, the strong search capability of this strategy can weaken the interference of local extrema on the PSO algorithm, thereby enhancing its search capability. The specific process is as follows:

Step 1: Initialize the population, initialize parameters, and initialize the velocity and position of the particles.

Step 2: Use the chaotic opposition-based strategy with Formulas (15) and (16) to generate a new population. Calculate the fitness values of the initial population and the two strategies, and select the top *N* optimal populations from them.

Step 3: Calculate fitness using a penalty function, and adaptively adjust the values of ω, $c_{1}$, and $c_{2}$ according to (17), (18), and (19). In each iteration step, select the solution with the highest probability as the optimal solution.

Step 4: Adjust the velocity of particles according to Formula (12), and perform one round of iterative optimization process according to Formula (13).

Step 5: Update the best position of individual particles and the best position of global particles according to (14).

Step 6: According to the greedy algorithm Formula (21), determine whether the new solution generated by the adaptive Cauchy–Gaussian mutation strategy Formula (20) should replace the global optimal solution.

Step 7: Determine if the stopping criteria for iteration have been met. The stopping criteria for this algorithm are set as follows: reaching the maximum number of iterations of 200, when the improvement in the objective function is less than the threshold (1 × 10^−8^), or if there is no change in the objective function value after several consecutive iterations (55 iterations in this algorithm), then the stopping criteria are met. If not met, return to Step 3.

Step 8: Terminate the algorithm and output the optimal particle.

**Figure 1:**
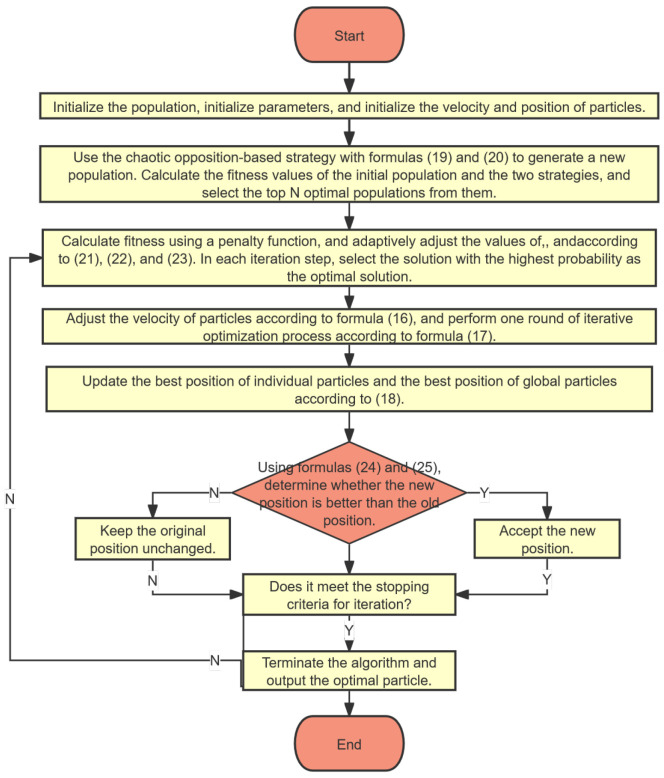
Flowchart of the MSPSO algorithm.

The flowchart of the MSPSO algorithm is shown in Figure 1:

## 4. Simulation Results of the Algorithm

### 4.1. Simulation of Emissivity Algorithms for Six Common Trend Materials

Xing conducted simulation experiments on six target materials and depicted the trends in emissivity variation in Ref. [21]. These materials exhibited six different emissivity change patterns, including increasing, decreasing, increasing, increasing, then decreasing, “W”-shaped, and “M”-shaped, thereby broadly representing the emissivity characteristics of most materials. This paper conducted simulations on the aforementioned six materials based on the MSPSO algorithm. At an actual temperature of 1800K, inversion calculations were performed on the six emissivity models, with a reference temperature of 1600K set. For easy distinction, these six materials were labeled as A to F. In this study, the multi-spectral pyrometer used is equipped with eight spectral channels, each corresponding to effective wavelengths of 0.4, 0.5, 0.6, 0.7, 0.8, 0.9, 1.0, and 1.1 µm, respectively. Using Ref. [21] and the wavelength and emissivity data provided for the six materials in the text, this paper utilizes Equation (2) to inversely calculate the corresponding voltage values, which serve as ideal reference values for evaluating the effectiveness of the algorithm. Table 1 lists the target emissivity data for these six model materials in detail.

During the solution process, the MSPSO algorithm performs a random search within the possible solution space based on the fitness values of the objective function. The algorithm utilizes feasibility principles to handle constraints, enhancing its accuracy and efficiency. However, if the feasible region is smaller, the search efficiency may be affected. Through multiple experimental simulations, we have found that the ideal range for emissivity should be between 0.1 and 0.9. By transforming the inequalities into a specific form, we can obtain a set of inequalities represented as $\left\{\begin{array}{cc} \varepsilon \left(\lambda_{i},T\right) & >0\\ -\varepsilon \left(\lambda_{i},T\right) & >-1 \end{array}\right.$.

**Figure 2:**
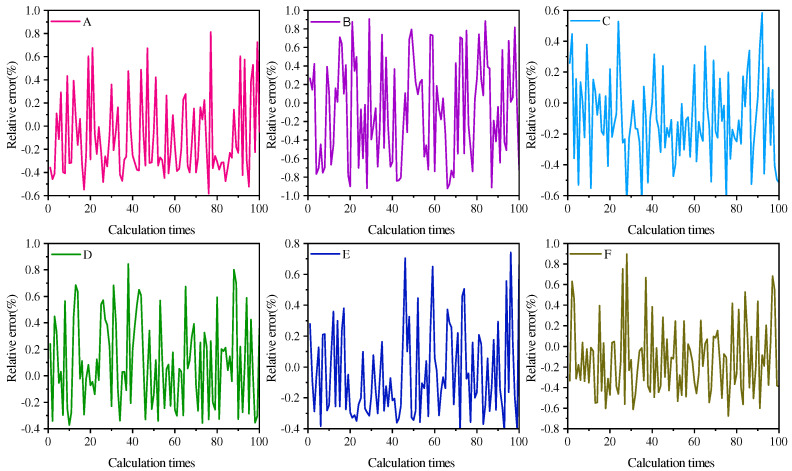
Calculate the relative error of temperature for six models using the MSPSO algorithm.

For this paper, the population size is established at 30, and the maximum iteration count is set to 200. Next, we follow the process outlined in Figure 1 to process the data model defined by Formula (11). The objective function constructed in this study is a complex multi-dimensional function with multiple local optima. Although this function has only one global minimum point, due to the inevitable errors in the data used for optimization (i.e., emissivity and signals), it is difficult to ensure that the function can accurately converge to the global minimum even if the algorithm performs exceptionally well. The global optimal solution of this function is unique, but a multitude of local optimal solutions accompany it. During each iteration of the algorithm, the initial solution is generated randomly and differs from one iteration to the next. To reduce errors, we adopt a method of multiple calculations. Using the MSPSO algorithm, we conduct 100 repeated calculations and harness parallel computing technology to enhance the algorithm’s execution speed. The average results of the inverted temperatures are listed in Table 2. In Figure 2, we present the relative error of each calculation at a temperature of 1800K.

According to the data presented in Table 2, under the condition of a true temperature of 1800 K, the maximum error in the inverted temperature of the measured target is only 2.74 K, with a corresponding maximum relative error of 0.26%. These calculation results fully demonstrate the high accuracy of the algorithm.

### 4.2. Comparison with Added Noise

**Figure 3:**
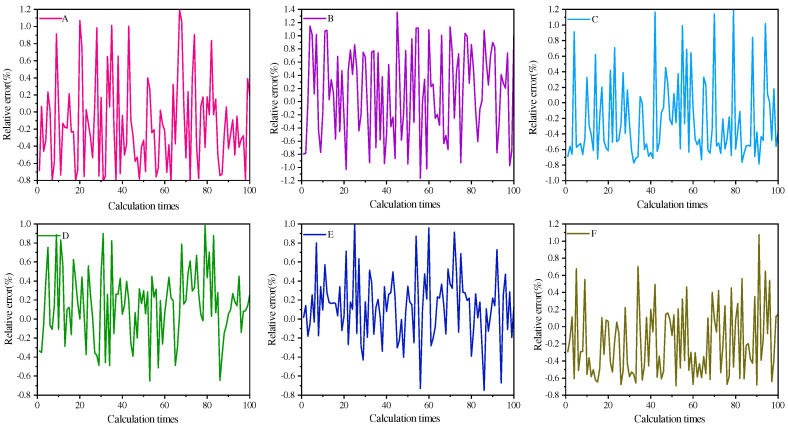
Calculate the relative error of temperature for six models using the MSPSO algorithm (with 5% random noise).

In real-world scenarios, the data voltage values we obtain are subject to noise errors. Therefore, we add 5% random noise to the voltage signal in Formula (4) to verify the noise resistance capability of the MSPSO algorithm. Through simulation experiments, we obtain the average results of the inverted temperatures, with the detailed data presented in Table 3. Additionally, we calculate the relative errors, and the results are shown in Figure 3.

After adding 5% random noise, by examining Table 3, we observe that the inversion results are roughly the same as those without noise, and even the inversion accuracy on some materials is improved. This fully demonstrates the excellent noise resistance capability of the MSPSO algorithm. By comparing Figure 2 and Figure 3, we can clearly see that the MSPSO algorithm exhibits outstanding stability and is expected to be widely used in industrial and other fields in practice.

### 4.3. Comparison of Algorithm Performance

To visually compare the performance of the genetic algorithm before and after improvement, we conduct a detailed comparative analysis of the two algorithms in terms of inversion emissivity, computation time, temperature error, and algorithm stability, based on the six emissivity model data in Table 1, under the same simulation environment.

**Figure 4:**
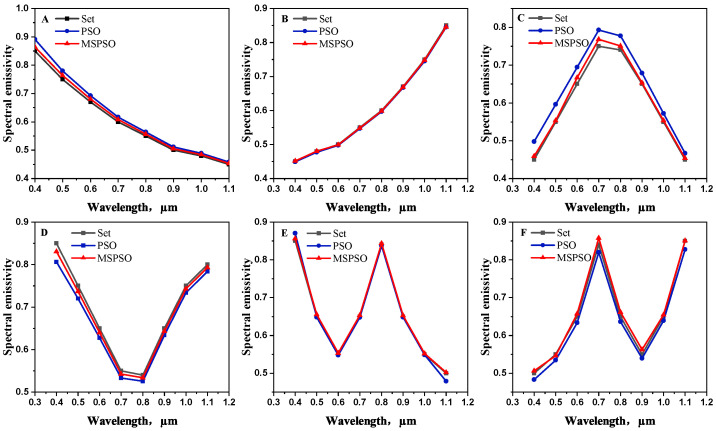
Comparison of emissivity calculated by PSO and MSPSO algorithms with true values (the six subfigures (**A**–**F**) represent the emissivity trends of six different materials.).

**Figure 5:**
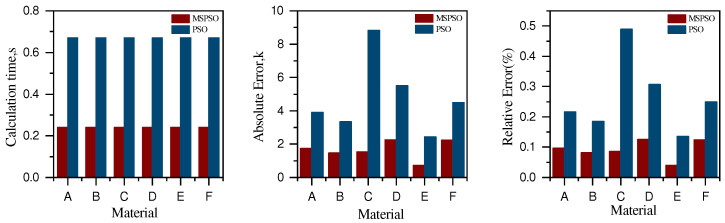
Comparison of computation time, absolute temperature error, and relative temperature error for six models before and after algorithm improvement.

Table 4 shows that the algorithm’s speed is increased by 64%, its accuracy is doubled, and its stability is also slightly improved. This is mainly attributed to the MSPSO algorithm optimized by multiple strategies. Through more uniform initialization by the chaos algorithm, particles are distributed throughout the entire solution space to forage, which makes it easier for particles to find the global optimal position. On the other hand, the Cauchy–Gaussian strategy perturbs particles trapped in the local optima, enabling them to jump out of their current positions and search for the truly optimal position. Adaptive parameter improvements also enable the algorithm to converge faster towards the global optimal position, accelerating the algorithm’s convergence time. Figure 4 and Figure 5 further demonstrate the superiority of the MSPSO algorithm over its predecessor.

From Figure 4, it can be seen that the trend of the emissivity inversion results of MSAPSO aligns significantly better with the true distribution than that of PSO. Figure 5 further demonstrates the superiority of the improved algorithm. It has significant advantages in terms of high accuracy, enhanced reliability, guiding material design, and reducing experimental costs, making it of great importance for material research and applications.

**Figure 6:**
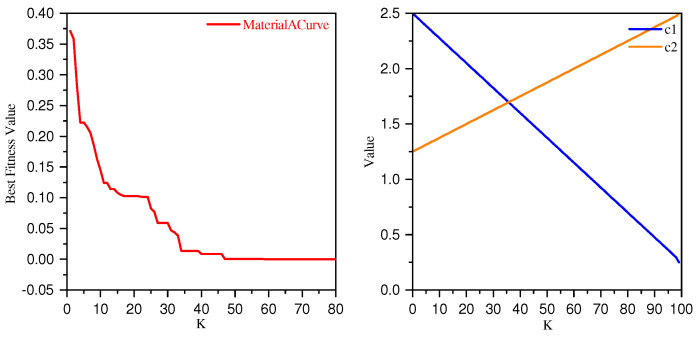
The figure shows the convergence of the objective function and the changes in parameters $c_{1}$ and $c_{2}$.

Additionally, we create a graph showing how $c_{1}$ and $c_{2}$ change with the number of iterations *k*. This graph visually represents the trend of $c_{1}$ and $c_{2}$ over time. Furthermore, we plot the convergence graph of the objective function to compare the impact of $c_{1}$ and $c_{1}$ on the convergence of the objective function. The specific graph is shown in Figure 6:

From the situation depicted in the figure, it can be seen that the objective convergence value gradually approaches zero as $c_{1}$ and $c_{2}$ change, proving the effectiveness of the parameter settings for $c_{1}$ and $c_{2}$. The convergence of the objective function shown in the figure indicates that the algorithm proposed in this paper tends to stabilize at certain stages of iteration, followed by a significant reduction in the objective function value. This phenomenon demonstrates the effectiveness of the proposed local escape algorithm.

## 5. Experimental Verification

To fully verify the accuracy of the MSPSO algorithm and its effectiveness in practical applications, we conduct practical application tests on the algorithm. Specifically, we apply it to the measurement of silicon carbide samples and tungsten samples to verify the effectiveness of the algorithm in actual production and daily life [29].

### 5.1. Experimental Preparation

**Figure 7:**
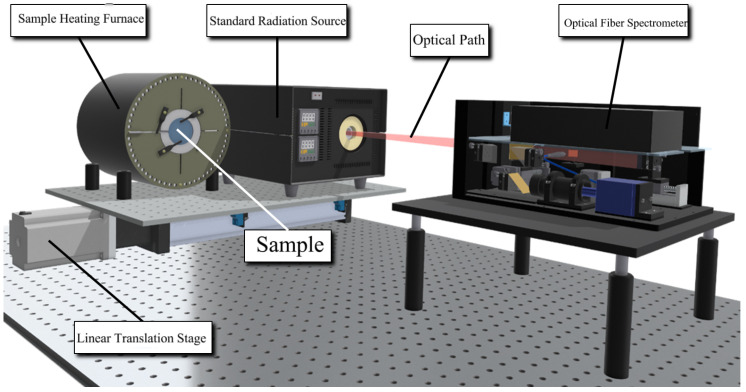
The 3D view of the emissivity measuring apparatus.

We use a rapid emissivity measurement device in the laboratory to detect the radiation signal of silicon carbide samples. The measurement system includes a fiber optic spectrometer (Idea Optics NIR25S), a halogen lamp calibration source, a high-temperature rangefinder, a linear displacement stage, an optical path system, and a host computer. The prototype features a dual-layer structure: the upper layer houses the measurement apparatus, while the lower layer contains the radiation signal capture and optical path calibration devices, connected via optical fibers. The prototype detector operates within a spectral range of 0.9–2.5 μm (near-infrared) and is capable of measuring temperatures within a distance of 0.5 to 3 m. The FWHM (Full Width at Half Maximum) of the channels in the fiber optic spectrometer is 3 nm. The spectrometer uses a two-stage thermoelectric-cooled InGaAs sensor with a spectral range of 0.9–2.5 μm and a signal-to-noise ratio (SNR) of 7500:1. The integration time is adjustable between 1 ms and 200 ms. The halogen lamp serves as a calibration light source, providing a spectrum that closely resembles blackbody radiation. With a color temperature of 2800 K, it covers an effective wavelength range of 360–2500 nm, ensuring accurate calibration during online measurements. This light source features high signal output stability (stability of less than 0.15% per hour), compact size, and ease of integration. The layout of the experimental equipment can be referred to in Figure 7.

A Proportional–Integral–Derivative (PID) temperature controller is used to control the blackbody temperature, with the temperature control system achieving a precision of 0.1 K. In the experiment, the blackbody temperature is set to 923 K, and the heating temperatures for the silicon carbide are set to 973 K, 1023 K, and 1073 K, respectively. An S-type thermocouple is used to monitor temperature fluctuations, while a calibrated K-type thermocouple is fixed in a small hole at the edge of the sample surface to monitor the sample temperature in real-time, with a maximum temperature of up to 1200 K. Once the surface temperature of the silicon carbide stabilizes, its radiation intensity is collected using a fiber optic spectrometer. The emissivity of the silicon carbide is determined by calculating the ratio of the sample’s voltage to the blackbody’s voltage. The measurement error of the equipment is controlled within 1–2%. In the experiment, measurement errors are ignored, and only the computational errors of the algorithm presented in this paper are considered.

Based on references [29,30,31], we can estimate the emissivity of silicon carbide and tungsten within a given wavelength and temperature range. After stabilizing the surface temperature of silicon carbide, we can measure its radiation intensity using an optical fiber spectrometer and combine this with data from the references to more accurately determine the emissivity range of both materials. Table 5 shows the measured spectral emissivity values of silicon carbide and tungsten at these wavelengths.

In the measurement of the tungsten sample, due to the limitations of the instruments, we refer to the content of references [29,30] and calibrate the blackbody temperature to 2000 K. We determine the emissivity range of tungsten within the specified wavelength range and calculate the corresponding voltage values based on these emissivities. Finally, the emissivity of tungsten is shown in Table 6.

### 5.2. Experimental Results

The following presents our inversion results for determining the emissivity and temperature of silicon carbide and tungsten. Although the temperature values calculated by the MSPSO algorithm vary each time, the results are all quite close to the true values. The inversion results listed below are based on the average of 100 repeated calculations. To enhance the computational efficiency and inversion accuracy, during the execution of the algorithm, we adjusted the feasible domains for the two samples to [0.5, 0.99] and [0.01, 0.5], respectively. Furthermore, this paper offers a comprehensive comparison between the proposed algorithm, the GIM-EPF algorithm, and the BP algorithm introduced by Xing, which combines the generalized inverse matrix with the long short-term memory neural network [19,21]. Through the analysis and comparison of these three algorithms, the aim is to evaluate their performance and applicability in specific tasks. The results are presented in Table 7 and Table 8.

The inversion results of silicon carbide using the MSPSO algorithm are detailed in Table 7. As is evident from the table, for silicon carbide material, the absolute error in the calculated temperature remains within 3 K, the relative error is less than 0.4%, and the average calculation time is merely 0.29 s. In contrast, for tungsten material, the detailed inversion results are presented in Table 8, where the absolute error is maintained within 7 K, the relative error does not exceed 0.3%, and the average calculation time is 0.38 s. Compared to the other two data processing algorithms, the algorithm proposed in this paper demonstrates overall superiority over the other two algorithms at three different temperatures for both silicon carbide and tungsten.

**Figure 8:**
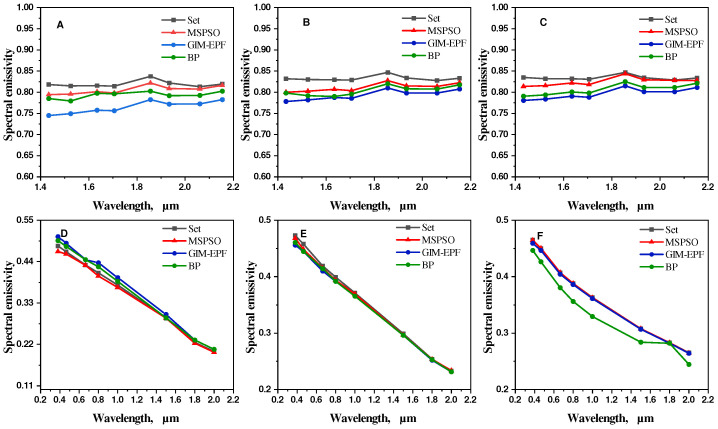
Comparison of measured spectral emissivity values with calculated values from three algorithms for two sample materials. Specifically, (**A**–**C**) represent silicon carbide, while (**D**–**F**) represent tungsten.

Furthermore, as shown in Figure 8, we compare the results obtained from the three algorithms with the actual emissivity trends. The trends presented in the figure clearly demonstrate that the spectral emissivity trends of the two samples obtained by inversion using the algorithm proposed in this paper are generally consistent with the actual distribution. Although the GIM-EPF algorithm and the BP neural network method show consistency in predicting the trends of material emissivity, their prediction performance is not satisfactory in some temperature ranges. In contrast, the algorithm proposed in this paper exhibits superior performance, surpassing the GIM-EPF algorithm and the BP neural network method. Therefore, we can conclude that the proposed MSPSO algorithm has good application potential in practical measurements of temperature and emissivity.

## 6. Conclusions

Based on the inherent relationship between the ratio of adjacent wavelength radiation signals and emissivity, we have innovatively proposed a novel data processing model for multi-spectral thermometers. Building upon this model and guided by nongradient optimization theory, we have further developed a new data processing algorithm named MSPSO. One significant advantage of this algorithm is that it does not require a preset spectral emissivity model or careful selection of an appropriate initial emissivity model, greatly simplifying the application process. Through a series of simulation experiments, we comprehensively model six different types of emissivity material models. The results show that the MSPSO algorithm can efficiently retrieve the true temperature and spectral emissivity with a relative error of less than 0.3%. Furthermore, in practical validation using silicon carbide and tungsten as experimental materials, the temperature relative error of the MSPSO algorithm is strictly controlled within 0.4%. Additionally, the computation time of this algorithm is extremely fast, within 0.4 s, which fully demonstrates its efficiency in practical applications. In summary, the data processing method we have constructed not only eliminates the need for an initialized spectral emissivity model but also ensures accurate and efficient temperature estimation, making it highly suitable for high-temperature real-time measurements in industrial settings. Moreover, since our algorithm does not have specific requirements for the functional form, it provides new optimization ideas and directions for future research on more complex and reliable multi-spectral temperature measurement models.

## Figures and Tables

**Table 1:** Emissivity of six model materials at various wavelengths [21].

Target	0.4 μm	0.5 μm	0.6 μm	0.7 μm	0.8 μm	0.9 μm	1.0 μm	1.1 μm
A	0.85	0.75	0.67	0.60	0.55	0.50	0.48	0.45
B	0.45	0.48	0.50	0.55	0.60	0.67	0.75	0.85
C	0.45	0.55	0.65	0.75	0.74	0.65	0.55	0.45
D	0.85	0.75	0.65	0.55	0.54	0.65	0.75	0.80
E	0.85	0.65	0.55	0.65	0.84	0.65	0.55	0.50
F	0.50	0.55	0.65	0.84	0.65	0.55	0.65	0.85

**Table 2:** Simulate the average result of temperature using the MSPSO algorithm.

T = 1800 K	A	B	C	D	E	F
Inversion Temperature (K)	1798.25	1801.47	1798.45	1802.24	1799.27	1797.77
Absolute Error (K)	1.74	1.47	1.54	2.74	0.72	2.22
Relative Error (%)	0.11	0.08	0.09	0.26	0.04	0.12

**Table 3:** Average result of temperature simulation using MSPSO algorithm (with 5% random noise).

T = 1800 K	A	B	C	D	E	F
Inversion Temperature (K)	1796.97	1803.3	1795.69	1802.17	1802.44	1796.50
Absolute Error (K)	3.02	3.33	4.30	2.17	2.44	3.49
Relative Error (%)	0.17	0.19	0.23	0.12	0.14	0.21

**Table 4:** Simulation results of average temperature before and after improvement in genetic algorithm.

Method	Absolute Error (K)	Relative Error (%)	Calculate Time (s)
PSO	8.81	1.48	0.67
MSPSO	1.54	0.12	0.24

**Table 5:** Spectral emissivity data of silicon carbide obtained via optical fiber spectrometer measurement.

				Wavelength (μm)				
TEMP (K)	1.434	1.525	1.635	1.706	1.856	1.934	2.061	2.154
973	0.8179	0.8152	0.8155	0.8145	0.8375	0.8217	0.8137	0.8193
1073	0.8179	0.8152	0.8155	0.8145	0.8375	0.8217	0.8137	0.8193
1073	0.8316	0.8298	0.8148	0.8287	0.8467	0.8335	0.8276	0.8331

TEMP (K) is used as an abbreviation for temperature (K).

**Table 6:** Spectral emissivity data of tungsten obtained through optical fiber spectrometer measurement.

				Wavelength (μm)				
TEMP (K)	0.380	0.467	0.380	0.800	1.000	1.500	1.800	2.000
2200	0.482	0.466	0.431	0.410	0.378	0.290	0.225	0.201
2800	0.473	0.458	0.419	0.399	0.371	0.299	0.254	0.233
3400	0.465	0.450	0.407	0.388	0.363	0.308	0.283	0.265

TEMP (K) is used as an abbreviation for Temperature (K).

**Table 7:** Inversion results of temperature of silicon carbide material by three algorithms.

Algorithm	Sample Temperature (K)	Inversion Temperature (K)	Absolute Error (K)	Relative Error (%)	Calculation Time (s)
MSPSO	973	976.01	3.01	0.31	0.29
	1023	1024.96	1.96	0.19	0.29
	1073	1074.24	1.24	0.11	0.29
GIM-EPF	973	982.18	9.18	0.94	3.67
	1023	1027.89	4.89	0.47	3.67
	1073	1077.67	4.67	0.44	3.67
BP	973	979.47	6.47	0.66	-
	1023	1027.34	4.34	0.42	-
	1073	1070.63	2.37	0.22	-

Since the training of neural network algorithms takes a long time, this will not be recorded in the table.

**Table 8:** Inversion results of temperature of tungsten material by three algorithms.

Algorithm	Sample Temperature (K)	Inversion Temperature (K)	Absolute Error (K)	Relative Error (%)	Calculation Time (s)
MSPSO	2200	2206.46	6.46	0.29	0.38
	2800	2806.77	6.77	0.24	0.38
	3400	3401.05	1.05	0.03	0.38
GIM-EPF	2200	2184.48	15.51	0.70	3.97
	2800	2808.70	8.70	0.31	3.97
	3400	3404.44	4.44	0.13	3.97
BP	2200	2210.17	10.17	0.46	-
	2800	2802.97	2.97	0.11	-
	3400	33413.45	13.45	0.39	-

Since the training of neural network algorithms takes a long time, this is not recorded in the table.

## Data Availability

The data presented in this study are available on request from the corresponding author.
